# Capturing the cloud of diversity reveals complexity and heterogeneity of MRSA carriage, infection and transmission

**DOI:** 10.1038/ncomms7560

**Published:** 2015-03-27

**Authors:** Gavin K. Paterson, Ewan M. Harrison, Gemma G. R. Murray, John J. Welch, James H. Warland, Matthew T. G. Holden, Fiona J. E. Morgan, Xiaoliang Ba, Gerrit Koop, Simon R. Harris, Duncan J. Maskell, Sharon J. Peacock, Michael E. Herrtage, Julian Parkhill, Mark A. Holmes

**Affiliations:** 1 Department of Veterinary Medicine, University of Cambridge, Cambridge CB3 0ES, UK; 2 Department of Genetics, University of Cambridge, Downing Street, Cambridge CB2 3EH, UK; 3 Wellcome Trust Sanger Institute, Hinxton CB10 15A, UK; 4 Department of Farm Animal Health, Faculty of Veterinary Medicine, Utrecht University, Utrecht 3584 CL, The Netherlands; 5 Department of Clinical Medicine, University of Cambridge, Cambridge CB2 0QQ, UK

## Abstract

Genome sequencing is revolutionizing clinical microbiology and our understanding of infectious diseases. Previous studies have largely relied on the sequencing of a single isolate from each individual. However, it is not clear what degree of bacterial diversity exists within, and is transmitted between individuals. Understanding this ‘cloud of diversity’ is key to accurate identification of transmission pathways. Here, we report the deep sequencing of methicillin-resistant *Staphylococcus aureus* among staff and animal patients involved in a transmission network at a veterinary hospital. We demonstrate considerable within-host diversity and that within-host diversity may rise and fall over time. Isolates from invasive disease contained multiple mutations in the same genes, including inactivation of a global regulator of virulence and changes in phage copy number. This study highlights the need for sequencing of multiple isolates from individuals to gain an accurate picture of transmission networks and to further understand the basis of pathogenesis.

## Abstract (web summary)

Populations of bacterial pathogens can be diverse within colonized individuals. Here, the authors sequence the genomes of methicillin-resistant *Staphylococcus aureus* isolated from staff and animal patients at a veterinary hospital and show considerable within-host diversity that can rise and fall over time.

## Graphical abstract

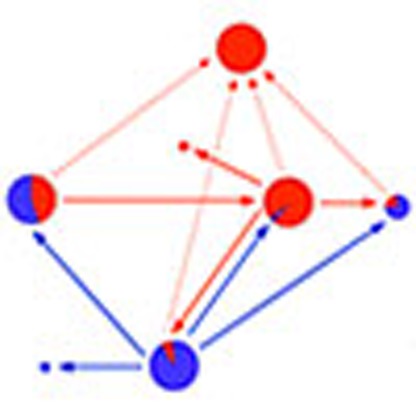

The use of rapid whole-genome sequencing in clinical microbiology is showing considerable potential for the diagnosis, characterization and surveillance of pathogens^1^,^2^,^3^. The power of genome sequencing to identify outbreaks and transmission events and to track the source of bacterial pathogens has been exemplified by a number of studies and will become an invaluable tool to inform hospital infection control, particularly in view of the increasing problems posed by multidrug-resistant pathogens^4^,^5^,^6^,^7^. However, studies to date have largely relied upon sequencing of single colonies from individual hosts, while recent data indicate within-host populations of bacterial pathogens may be heterogeneous. Recent studies on sequencing multiple individual isolates from *Staphylococcus aureus* carriers have shown that individual colonies that differ by up to 40 single-nucleotide polymorphisms (SNPs) can be isolated from a single colonized individual^5^,^8^,^9^. Currently, little data are available about how this within-host ‘cloud of diversity’ fluctuates temporally, in newly, or long-term colonized or infected individuals. Importantly, no data are available regarding the degree of bacterial diversity that is transferred from one individual to another in a transmission event.

To further understand the cloud of diversity that exists within colonized individuals and during transmission, we undertook deep sequencing of a methicillin-resistant *S. aureus* (MRSA) ‘outbreak’ at a veterinary hospital involving both staff and animal patients. We show that there is considerable within-host diversity during carriage and infection, which may rise and fall over time, and can include multiple genotypes. Our data also provide new insights into the degree of diversity transmitted between individuals. Finally, we highlight the need for sequencing of multiple isolates for the accurate determination of transmission networks.

## Results

### Investigation of MRSA transmission in a veterinary hospital

**Figure 1:**
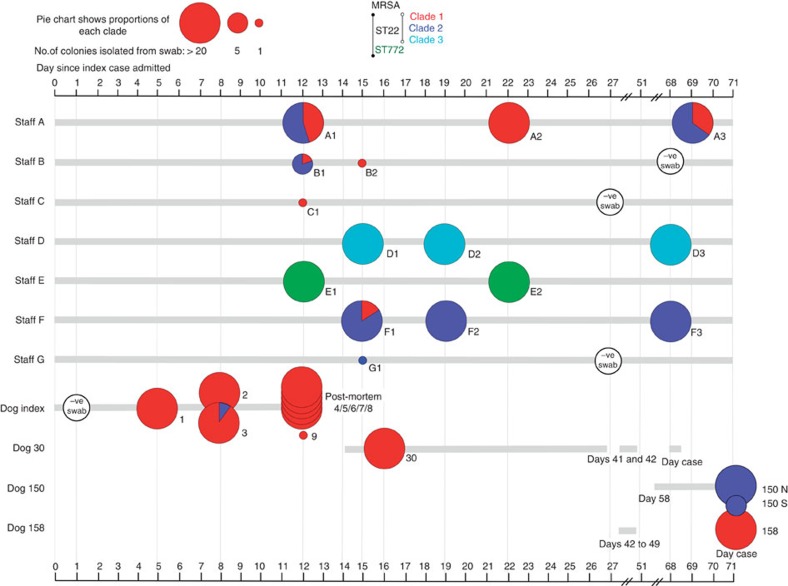
Time line of the presence and swabbing dates of staff members and animal patients in the veterinary hospital. Each circle represents an individual swab, the size of the circle is proportional to the number of colonies recovered after culture and the colours represent the relative proportions of a particular clade in the colonies assessed by whole-genome sequencing.

The index case was a 4-year-old German Shepard dog admitted to a veterinary hospital with suspected toxic epidermal necrolysis manifesting in an open abdominal wound. A wound swab on admission produced a positive culture for *Pasteurella multocida*, which was fully susceptible to all antibiotics tested. Five days after admission, a second wound swab grew MRSA. The animal’s condition deteriorated and it died 11 days after admission. A post mortem was performed, confirming the cause of death as *S*. *aureus* sepsis. Additional swabs of the index case taken at 8 days and samples taken at post-mortem were all positive for MRSA. To identify the potential source of infection and other transmission events, we screened the hospital staff and animal patients for MRSA. A total of 97 members of staff and 158 animal patients from the veterinary hospital were screened for MRSA carriage. Any MRSA-positive staff members were re-swabbed at intervals to identify persistent carriers (Fig. 1). Seven staff members (Staff A–G) and three more animal patients (Dogs 30, 150 and 158) were positive for MRSA (prevalence of 7.2% and 6.8%, respectively). Six of the seven MRSA-positive staff members (Staff A, B, C, D, F and G) were involved in the direct care of the index case, whereas all three of the MRSA-positive animals first entered the hospital after the death of the index case (Fig. 1). Therefore, the availability of dense sampling of both staff and animals taken both during the initial case and in the time immediately afterwards presented the opportunity to investigate bacterial diversity present within and potentially transferred between each colonized host.

### Genomic characterization of MRSA populations

**Figure 2:**
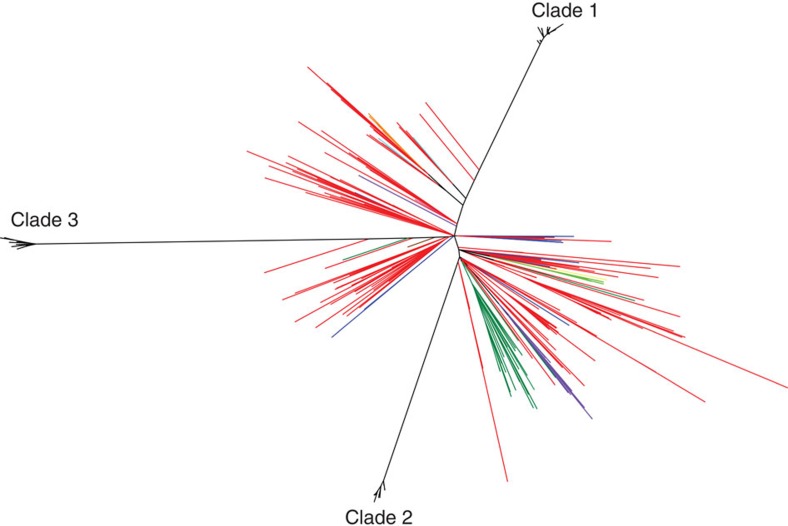
Phylogenetic relationship of three distinct clades of ST22 isolates from the veterinary hospital in comparison to a global collection of ST22 isolates^13^,^39^. Isolates are coloured according to country of origin. UK=red, Germany=dark green, Australia=dark blue, Portugal=Purple, Denmark=light blue, Czech republic=light green, Sweden=brown, Nigeria=maroon, Hungary=yellow, Singapore=yellow and New Zealand=pink.

Whole-genome sequencing was carried out on 20 separate colonies (or all the colonies grown if the total was less than 20) from each MRSA-positive swab from the staff and animals (Fig. 1). Multilocus sequencing types extracted from the genome sequence identified the presences of two different sequence types: ST22 (EMRSA-15)^10^ and ST772 (Bengal Bay clone)^11^,^12^. However, ST772 was only found in a single member of staff, whereas ST22 was isolated from the index case, five members of staff (staff A, B, C, D and F) and three animal patients (dogs 30, 150 and 158; Fig. 1). A phylogenetic tree constructed using SNPs present in the core genome, identified that three distinct clades of ST22 were circulating in the veterinary hospital (clades 1, 2 and 3) separated by >180 SNP from each other (Figs 1 and 2). The majority (141 of 143) of the isolates from the index case were clade 1, suggesting that organisms from this clade were causing the pathology. A swab taken from the axilla in the index case was also positive for two clade 2 isolates (Fig. 1). Co-colonization with isolates from both clades 1 and 2 was also seen in three other staff members (staff A (samples: A1 and A3), staff B (B1) and staff F (F1) in Fig. 1). In staff member A, the relative proportions of the two populations fluctuated over the three sample points taken over 57 days (% proportions of clade 1:clade 2 at day 12=45:55, at day 22=100:0 and at day 69=35:65; Fig. 1). Staff member F’s first swab also produced colonies from both clade 1 and 2 (clade 1:clade 2=15:85), although their second and third swabs only produced isolates from clade 2 (staff F2 and F3 in Fig. 1).

### Temporal changes in within host diversity in persistent MRSA carriers

**Figure 3:**
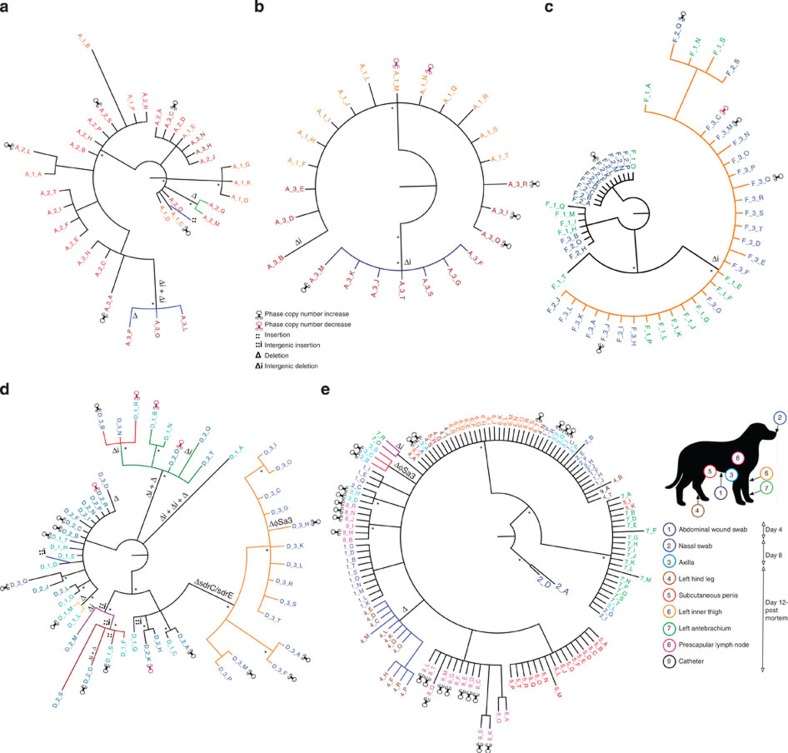
Individual phylogenetic trees for Staff member A, F, D and Index Dogs isolates. Bayesian phylogenetic trees generated using SNPs, indels and the presence/absence mobile genetic elements (MGEs) to inform the topology for Staff member A’s: (**a**) Clade 1 isolates and (**b**) Clade 2 isolates, (**c**) Staff member F’s (Clade 2 isolates), (**d**) Staff member D (Clade 3 isolates) and (**e**) the Index Dog (Clade 1 isolates). Coloured branches represent strains that share the presence/absence of an indel/MGE. Some indels/MGEs are not coloured if they are not topologically informative, they share their presence/absence with another indel or they are the ancestral state. Ancestral states are described at the root and at some nodes of the phylogeny. All nodes present in the trees have >0.5 support and starred nodes have >0.95 support. The presence of insertions, deletions and changes in phage copy numbers are shown on branches or individual taxa. The figure of the dog shows the location of the samples, the colours of the circles marked on the representation of the dog match those on the tip labels of the tree in **e**.

**Figure 4:**
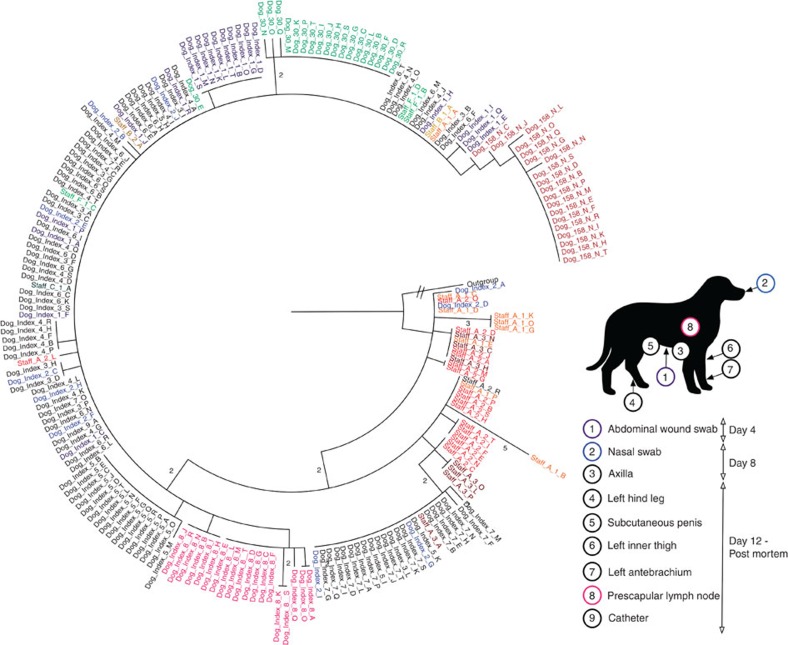
Maximum likelihood tree generated from SNPs in the core genome for isolates from Clade 1. Numbers above the branches indicate the number of differentiating SNPs. Isolates from repeated swabs from same individual staff member or animal are numbered sequentially and coloured, with darker tones representing later isolates. The location and time of the swabs from the index case are highlighted in the graphical representation of a dog.

We analysed the MRSA populations in the three staff members who were persistent carriers to define changes in diversity over time. The 34 clade 1 isolates from staff member A were differentiated by a total of 22 SNPs, 4 deletions (two intergenic) and 1 insertion (Figs 3a and 4). Diversity was observed across the three time points, with both unique and identical isolates (defined henceforth as the same genome-type) in the second and third swabs compared with the first (Fig. 3a). The 24 clade 2 isolates from staff member A were differentiated by 11 SNPs and two intergenic deletions (Fig. 3b). The 11 isolates from swab one were identical except for one SNP variant, but the 13 isolates from swab two split into two distinct sub-clades (Fig. 3b). The second distinct sub-clade of seven isolates shared a common ancestor with the first clade and was differentiated by five SNPs.

**Figure 5:**
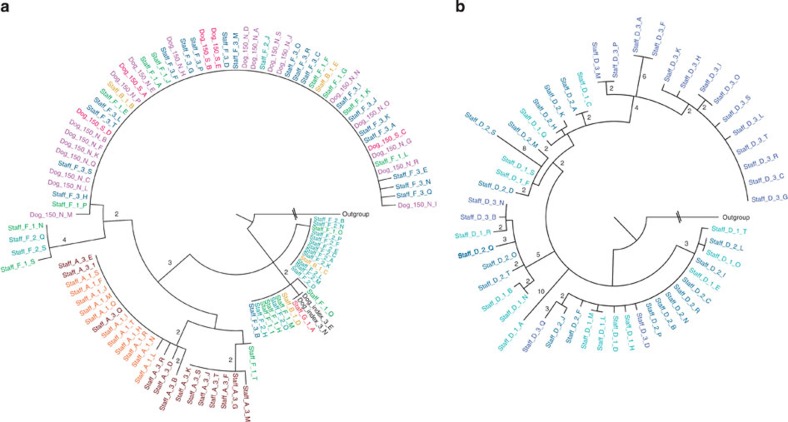
Phylogenetic trees of Clade 2 and Clade 3 isolates. Maximum likelihood trees generated from SNPs in the core genome for isolates from (**a**) Clade 2, (**b**) Clade 3. Numbers above the branches indicate the number of differentiating SNPs. Isolates from repeated swabs from same individual staff member or animal are numbered sequentially and coloured, with darker tones representing later isolates.

The clade 2 isolates from staff member F were differentiated by a total of 19 SNPs and a single intergenic deletion, with changes in diversity observed over time. The 16 isolates from swab one (Staff F1), taken on day 14 varied by 16 SNPs and a single intergenic insertion (Figs 3c and 5a). The second swab (Staff F2) taken on day 19 produced 16 isolates, 14 of which were identical to isolates from the first swab, whereas the isolates from swab three taken on day 69 (Staff F3) were more homogenous, with 16 of identical genome-type and three isolates each differing by only a single SNP (Fig. 3c and Supplementary Fig. 2). The predominant genome-type from swab three was detected at lower frequency in the two previous swabs, indicating a shift over time in the predominant colonizing population.

Staff member D was the only carrier of clade 3 isolates, which was more diverse than the other two clades combined (84 SNPs, 7 intergenic deletions, 6 deletions, 4 intergenic insertions and 1 insertion; Figs 3d and 5b). The higher diversity was confirmed by tests of pairwise diversity and Watterson’s theta (Supplementary Fig. 2). One explanation for this higher diversity is a higher mutation rate in clade 3 compared with clade 1 and 2, but this was not shown experimentally (Supplementary Table 1). Furthermore, maximum *a posteriori* estimates from the genome sequence data, which revealed that isolates from clade 3 had a mutation rate of 8.1 × 10^−6^ substitutions per nucleotide site per year (95% Bayesian credible intervals: 4.6 × 10^−6^–1.3 × 10^−5^), comparable to a previous measurements of ST22 population: 1.3 × 10^−6^ substitutions per nucleotide site per year (95% Bayesian credible intervals, 1.2 × 10^−6^ to 1.4 × 10^−6^)^13^. An estimate for time to most recent common ancestor was 179 days (95% highest posterior density (HPD): 177–288 days). The majority of the isolates (14 of 18) in the third swab were part of a distinct clade not seen in the first two swabs, although directly descended from populations that were (Fig. 3d). All 14 isolates in this clade had an 8.5-kb deletion between *sdrC* and *sdrE* (deleting *sdrC*, *sdrD* and *sdrE*) present in 14 isolates (Fig. 3d). A single isolate (Staff D_3_H) from this clade had also lost the φSa3 phage, which encodes modulators of the human innate immune response^14^,^15^. We also identified that all the isolates from staff member D had a premature stop codon (Trp76STOP) present in *agrC* (AgrC is the autoinducer sensor protein component of the accessory gene regulator (*agr*), a quorum sensing system and global transcriptional regulator of staphylococcal virulence^16^). The loss of *agr* function was confirmed by a lack of δ-haemolytic activity (a proxy for *agr* activity^17^; Supplementary Table 2 and Supplementary Fig. 1).

### Analysis of within host diversity during infection

We next analysed the isolates from the index case. A total of 141 isolates were differentiated by 24 SNPs. In all, 57 of the 141 (40%) isolates were genetically indistinguishable (the same genome-type) on the basis of core genome SNPs (isolates identical to Dog_Index_1_C in Fig. 3e). Another 53 isolates only differed by a single SNP from this population, meaning that 78% of isolates from the index case varied by a maximum of two core genome SNPs. All the isolates from the first MRSA-positive swab taken on day 5, were either the predominant genome-type (7 isolates) or belong to one of two single SNP sub-clades (3 and 10 isolates; Fig. 3e). The isolates from the nasal swab taken on day 8 (Dog Index 2) contained the most SNPs (nine SNPs between ten isolates) and were the most diverse sample from the index case (Supplementary Fig. 2). Clusters of isolates from individual anatomical sites were differentiated into sub-clades that descended from the predominant genome-type (Dog Index 1, 3, 4, 5, and 8 in Fig. 3e). Three isolates (Dog_Index_3B, C and S) from the axilla had also lost the φSa3 phage, interestingly, like in staff member D, two of three isolates that had lost φSa3 phage also had increased φSa2_HO 5096 0412_ copy number (Fig. 3e and Supplementary Fig. 4). Unlike the broad distribution of isolates with altered φSa2_HO 5096 0412_ copy number in staff A, F and D, all but two of the isolates from index case with an increased φSa2_HO 5096 0412_ number were phylogenetically distinct isolates from the axilla (Dog Index 3, 8 of 11 isolates) and the precapsular lymph node (Dog Index 8, 14 of 19 isolates; Fig. 3e and Supplementary Fig. 4). Furthermore, all isolates from the only invasive site sampled, the prescapular lymph node (Dog Index 8) were differentiated from the predominant genome-type of the index case by two different SNPs - present in the same gene: *agrR* (AgrR is the response regulator component of the accessory gene regulator (*agr*)^18^. The first SNP, present in 13 isolates, was a non-synonymous mutation causing a Ser202Asn substitution (isolates around Dog_Index_8_F in Fig. 3e). The second mutation, a premature stop codon (Lys236STOP) at the C-terminal end of AgrA was present in six isolates (isolates around Dog_Index_8_B in Fig. 3e). The loss of δ-haemolytic activity was confirmed in multiple isolates with both of these *agrA* mutations, demonstrating the mutations caused a loss of AgrA function (Supplementary Table 2 and Supplementary Fig. 1). Furthermore, two of the isolates (Dog_Index_8_S and K) with the S202N substitution had two further non-synonymous mutations (Gln1256Glu and Asn1329Asp) in the same gene: *fmtB*, a surface anchored protein associated with methicillin resistance^19^.

### Interpretation of transmission pathways for clade 1

Next we analysed the entire clade 1 data set to elucidate possible transmission events. The isolates from staff member A were consistently the most basal, with identical and unique basal isolates being present in all three swabs (Figs 3a and 4 and Supplementary Fig. 2). Furthermore, the isolates from staff member A’s first swab (Staff A1 in Figs 3a and 4) had the greatest pairwise diversity of all clade 1 samples (Supplementary Fig. 2a). For the index case, all isolates except for two basal isolates (Dog_Index_2_D and Dog_Index_2_A) in the nasal swab taken on day 8 were descended from the basal population present in the staff member A (Fig. 4). Directly basal isolates to the sub-clade of isolates from the left antebrachium (forearm; Dog_index_7 in Fig. 4) were present only in staff member A (Staff_A_2_N and C in Fig. 3). At days 12 and 22, staff member A also carried isolates that were either identical (Staff_A_1_A) or that differed by a single SNP (Staff_A_2_L) to the predominant genome-type in the index case (Fig. 4). Interestingly, all the isolates from the three other clade 1-positive staff members (Staff B, C and F) were identical to the predominant genome-type found in the index case. Whereas the two clade 1-positive animal patients dog 30 (positive on day 16) and dog 158 (positive on day 71–59 days after the death of the index case) were both colonized by distinct sub-populations of isolates that displayed very low levels of diversity (differing by up to 4 SNPs) and that descended from the predominant genome-type in the index case (Fig. 4 and Supplementary Fig. 2). Dog 30 also produced a single isolate identical to the predominant genome-type of the index case (Fig. 4).

### Evidence for transmission of clade 2 isolates

As for in clade 1, one staff member was persistently colonized with diverse basal isolates of clade 2, namely, staff member F (Fig. 5a and Supplementary Fig. 2). All the clade 2 isolates from staff member A (the other persistent carrier that was co-colonized with clades 1 and 2) from both positive swabs were descended from the basal population present in staff member F (Staff A1 and A3 in Fig. 5a). Furthermore, an isolate from staff member F (Staff_F_1_T) was directly basal to one of the two sub-clades from staff member A’s third swab (Fig. 5a). Dog 150, which was both nasally colonized and had wound infected by clade 2 isolates, first entered the veterinary hospital on day 58, 10 days before the third swab was taken from staff member F (Fig. 1). Dog 150 was populated with isolates that were identical to the predominant genome-type (or that differed by a single SNP) of isolates from staff member F in their third swab (Staff_F_3; Fig. 5a). In contrast to the homogeneity of clade 2 isolates seen in the dog 150, the four clade 2 isolates from staff member B (co-colonized with clade 1 and clade 2) differed by a total of ten SNPs (with two identical isolates from the predominant sub-clade seen in dog 150 and staff F3 in Fig. 5a). Despite the degree of diversity present being equivalent to staff member F, staff member B was only positive for clade 2 isolates on day 12 and was negative 2 days later, suggesting they were only a transient carrier (Figs 1 and 5a and Supplementary Fig. 2). Finally, the clade 2 isolates from staff member G (single isolate: Staff_G_1_A) and the index case (two isolates: Dog_Index_3_E and N) both differed by a single SNP from the genome-type of one of the basal clades that made up the majority of staff member F’s population at their first two swabs taken at the same time (Staff_F1 and Staff_F2 in Fig. 5a).

## Discussion

**Figure 6:**
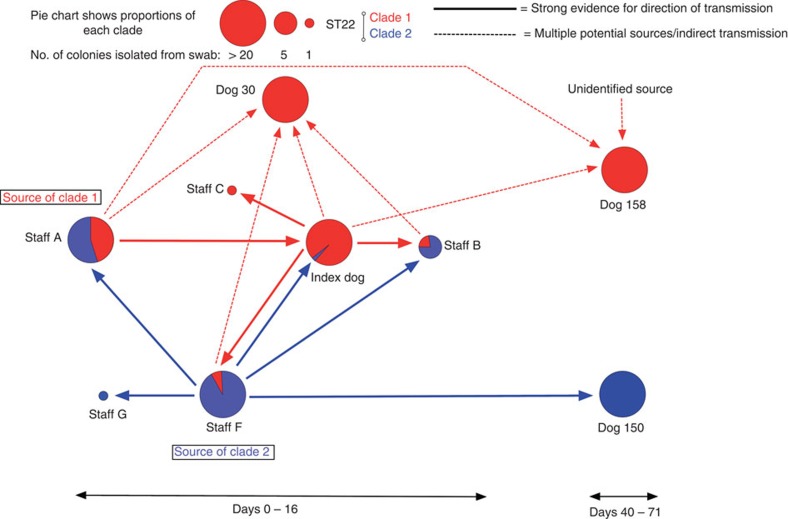
Summary of inferred transmission pathways. Figure shows the inferred transmission pathways between Staff members and Dogs based on combined genomic and epidemiological evidence. The size of the circle is proportional to the number of colonies recovered after culture from their first swabs. Except for the Index Dog where the number of Clade 2 isolates is not proportional to allow for visualisation. The colours represent the relative proportions of a particular clade in the colonies assessed by whole-genome sequencing.

We have used whole-genome sequencing of multiple individual colonies to investigate MRSA carriage and transmission among human staff and animal patients at a veterinary hospital. Using a combination of epidemiological and genome sequence data it is possible to elucidate the probable MRSA transmission events. For the clade 1 isolates (the cause of the index case infection), the most parsimonious explanation for the source was staff member A, because: (i) their isolates were consistently the most basal in the phylogenetic tree, (ii) the isolates in their first swab exhibited the greatest pairwise diversity, (iii) they possessed isolates representing the predominant genome types in the index case, (iv) they were the only known persistent carrier of clade 1 and (v) they were directly involved in care of the index case (Fig. 6). Similar reasoning suggests that for staff members B, C and F it was most likely that they acquired their isolate by transmission from the index case (Fig. 6). In the case of dog 30, either staff members A, B and F or the index case (indirectly through environmental transmission) might have been the source (Figs 4 and 6). For dog 158, although colonized by a population derived from the predominant genome-type of the index case, it first entered the hospital 32 days after the death of index case (Fig. 1). The only known carrier at this time was staff member A, who was still populated with basal isolates, therefore the chain of transmission is not clear (Fig. 6).

We also found that at the same time, a second distinct clone of ST22 (clade 2) had been transmitted between staff and animal patients. Staff member F was the most likely source of clade 2 as they were a persistent carrier, and were populated with the most diverse and basal isolates. Staff member A most likely acquired their clade 2 isolates from staff member F, as staff members F’s isolates were basal to the population in staff member A (Figs 5a and 6). For staff member B, the direction of transmission was less clear, as their four isolates were as diverse and representative of the population present in staff member F’s at approximately the same time (Staff B *cf*. Staff F1 and F2 in Fig. 5a and Supplementary Fig. 2). However, staff member B was not a persistent carrier, and most likely acquired a diverse population from staff member F (Figs 1 and 6). This clade was subsequently acquired by staff member G and the index case, again, most likely from staff member F as their isolates were identical to or differed by a single SNP to staff member F’s contemporaneous isolates. Clade 2 isolates were also found in dog 150. These isolates were identical or varied by a single SNP to the predominant genome type in staff member F’s third swab (Fig. 5a). Dog 150 first entered the veterinary hospital close to the time of staff member F’s third swab (Fig. 1) suggesting that staff member F was a likely source of the clade 2 isolates in dog 150 (Fig. 6).

This study yielded some interesting insights concerning within-host bacterial diversity during colonization and following transmission. Co-colonization by distinct *spa*-types has been described^20^ and here we provide evidence of colonization by separate sub-populations of the same multi-locus sequence type. The almost equal proportions of clade 1 and 2 isolates in staff member A’s initial swab indicate that the possibility of correctly identifying their involvement in clade 1 transmission using a single isolate chosen at random would have been only 45% (Staff A 1 in Fig. 1). The implications of this finding are not only limited to *S. aureus*, as co-infection or co-colonization by a number of bacterial and viral pathogens has been reported^10^,^21^,^22^,^23^,^24^,^25^,^26^,^27^,^28^.

The deep sequencing of temporally spaced samples identified that varying degrees of diversity are present in colonized individuals and that the composition of colonizing populations can shift dramatically, including becoming less diverse over time (as in the case of the third swab from Staff member F; Fig. 5a and Supplementary Fig. 2). This situation may be more complex than reported here using only nasal swabs, as variation in populations from different body sites has been reported for *S. aureus*^29^,^30^,^31^.

The extent of the diversity of *S. aureus* in host populations will be influenced by the amount of diversity transferred during a transmission event and by the duration between transmission event and sampling. The data presented here suggest that the populations present in post-transmission recipients can either be homogenous (Clade 1: Staff B, C, F, Dog 30 and 158, Clade 2: Staff G and Dog 150) or heterogeneous (Clade 1: Index case, Clade 2: Staff B) (Figs 4 and 5a). Of course, no data were available about the number of transmission events or their exact timing, or the nature of population bottlenecks occurring post transmission.

Three staff members (staff A, D and F) were persistent MRSA carriers but had different rates of transmission. Staff member D, although an active member of the clinical team looking after the index case, neither transmitted their clade (clade 3) nor acquired any of the other clades. Staff member A, who was the apparent source of clade 1 also carried clade 2, but did not transmit this clade. Behavioural characteristics, the nature of the contact, adherence to hygiene and aseptic technique, all may be expected to influence the likelihood of transmission and acquisition. Furthermore, biological characteristics of the individual bacterial lineages and hosts also likely influence the likelihood of transmission and colonization.

Studies using theoretical modelling have highlighted caveats in the use of sequencing data from single colonies alone to infer transmission^32^,^33^. Our data provide empirical evidence from a real world situation that supports the concerns raised by modelling work. The selection of 20 individual isolates to sequence was arbitrary; is there an optimal number of isolates? A rarefaction and extrapolation analysis of isolates obtained from the index case (Supplementary Fig. 3) suggests that there would be a near linear increase in the number of genomic variants obtained with increased sampling. One answer might be the use of shotgun metagenomics^34^,^35^,^36^,^37^ to assess the degree of diversity present in clinical samples (with microbial DNA enrichment^38^) or cultured isolates to provide an empirical basis for the selection of the number of colonies to sequence.

Previously, we have shown a shared population of CC22 MRSA circulates in humans, cats and dogs^39^. Here, we demonstrate direct transmission occurring in both directions, substantiating the view that ST22 has a broad host range and behaves as a nosocomial pathogen within a veterinary health-care setting just as it does within human hospitals^39^,^40^.

An investigation of mutations among isolates within the index case sheds light on the evolution of the pathogen over the course of infection. We identified that the population present in the lymph node of the index case all had one of two different mutations causing inactivation of the same gene: *agrA*. No other clade 1 isolates had the same mutations, suggesting they were present at very low frequency or they were generated *de novo* during infection. Selection of *agr* mutants by the presence of antibiotics (the index case was treated throughout its time in hospital) has been reported, probably due to the fitness cost of RNAIII expression, providing *agr* defective ‘cheaters’ a distinct fitness advantage^41^,^42^,^43^,^44^. *agr* dysfunction has also been identified as risk factor for persistent bacteremia in human patients and is associated with a poor clinical outcome^45^,^46^,^47^. Our findings, extending these observation to a canine host suggest that inactivation of the *agr* system might be advantageous for survival irrespective of the host species. The isolates from lymph node and axilla all had an increased copy number of φSa2_HO 5096 0412_. Given the proximity of the axilla to the prescapular lymph node this might suggest that the increase in phage copy occurred in the ancestral population in the axilla and that this population then seeded the invasive infection. Further work is required to investigate the role of both φSa2_HO 5096 0412_ and phage-copy number variation in *S. aureus* pathogenesis.

In conclusion, this study confirms the value of whole-genome sequencing in the epidemiological investigation of nosocomial transmission of MRSA while identifying potential pitfalls. It highlights the potential complexity of transmission networks and the potential limitations of obtaining sequencing data from a single isolate from a host. Although this study was performed in a veterinary context its results are highly relevant to any health-care setting.

## Methods

### Ethical review

The proposed study was submitted to the Ethical Review Committee at the Department of Veterinary Medicine at the University of Cambridge. It was approved on 22 April 2013 (CR88).

### MRSA screening and bacterial isolation

Sterile Ames media transport swabs (Medical Wire) were used to sample both anterior nares of staff members and animal patients. The personnel who conducted the swabbing and processing of samples were also swabbed and found to be negative for MRSA. Swabs were then inoculated into 4 ml of Müller Hinton broth with 6.5% NaCl and grown statically for 24 h at 37 °C in air. One hundred microlitres of broth were then plated onto MRSA Brilliance Agar 2 plate (Oxoid) and incubated for 24 h at 37 °C in air. Plates with no growth were incubated for a further 24 h to confirm the negative result. Putative MRSA were confirmed by PCR for *mecA* and *femB*^48^.

### Whole-genome sequencing

Genomic DNA was extracted from overnight cultures grown from single colonies in 5 ml of tryptic soy broth overnight at 37 °C using the MasterPure Gram Positive DNA Purification Kit. Illumina library preparation was carried out as described by Quail *et al*.^49^, and Mi-seq or Hi-seq sequencing was carried out following the manufacturer’s standard protocols (Illumina, Inc.). Nucleotide sequences been deposited in the European Nucleotide Archive (Supplementary Data set 1).

### Bioinformatic analysis and phylogenetics

Fastq files for the isolates were mapped against the ST22 MRSA reference genome HO 5096 0412 (EMBL accession code HE681097) using SMALT (http://www.sanger.ac.uk/resources/software/smalt/) in order to identify SNPs, as previously described (Supplementary Data sets 2, 3 and 4)^50^. SNPs located in mobile genetic elements or low-quality regions (insertions and deletions (indels)/low coverage/repeat regions) were identified by manual inspection and removed from the alignments (Supplementary Data sets 5, 6 and 7). High-quality SNPs were then manually inspected in the alignment and using BAM files mapped on the reference genome. Any isolates with large numbers of N’s (uncalled SNPs) in the alignment were removed from the analysis as potentially contaminated. The maximum likelihood tree was generated from the resulting SNPs in the core genome using RAxML^51^. Indels were identified as previously described^52^ (Supplementary Data sets 2, 3 and 4). Indels were manually assessed using BAM files mapped on the reference. Comparison of the mobile genetic content of the isolates was assessed by BLAST analysis against Velvet *de novo* assemblies^53^.

### Experimental measurement of mutation rate

The mutation rates for representative isolates from each clade were measured using the methods described by O’Neill and Chopra^54^. Briefly, three independent colonies were picked for three representative isolates distributed throughout the phylogeny of each ST22 clade (Clades 1, 2 and 3) and grown in 5 ml Isosensitest broth (Oxoid) at 37 °C at 200 r.p.m. until they reached an optical density of ~1 at 595 nm. One hundred microlitres of each culture was then serially diluted and plated onto either Iso-Sensitest agar plates (Oxoid) containing 4 × minimum inhibitory concentration of rifampicin (as determined by agar dilution^55^) or on Iso-Sensitest agar plates. Mutation rates were calculated as the number resistant colonies recovered on the rifampicin plates as a proportion of the total population as determined on Iso-Sensitest plates (Supplementary Table 1). A previously described *mutS*-knockout strain RN4220*mutS* and its isogenic wild-type RN4200 (ref. 54) were included as controls with mutation rates of 3.02(±0.13) × 10^−7^ and 3.19(±0.13) × 10^−6^, respectively, a 9.5-fold difference, and similar to that previously reported for these strains^50^.

### Haemolysis assay

A haemolysis assay was carried out as previously described^17^. A single colony of *S. aureus* RN4200 was streaked down the centre of a Columbia agar with sheep blood plate (Oxoid). Single colonies of representative isolates were then cross-streaked horizontally up to the RN4220. Plates were then incubated for 18 h at 37 °C followed by 6 h at 4 °C. Isolates that produced enhanced haemolysis in the area close to RN4220, which only produces β-haemolysin were scored as positive for production of δ-haemolysis (Supplementary Table 2). Representative photographs are shown in Supplementary Fig. 1.

### BEAST analysis

The presence of temporal signal was tested for separately in the isolates sampled from staff A, D, F and the index dog, through fitting a regression of root-to-tip genetic distance against sampling date using Path-O-Gen v1.4 (http://tree.bio.ed.ac.uk/software/pathogen/). Temporal signal was only judged as present in the isolates from staff D. For the samples from this individual, the fit of linear regression of root-to-tip distance against sampling time to the data had a large and positive correlation coefficient (*r*=0.68). For other individuals, this was not the case (staff A, *r*=0.11; staff F, *r*=−0.26; index dog, *r*=0.06). The date of the most recent common ancestor and the evolutionary rate were estimated using BEAST for isolates from staff D^56^. A HKY substitution model with a 4-category gamma distribution of rates and no partitioning of sites was used. Because of low levels of divergence, a strict molecular clock model and constant population size model were used. Estimates were similar to those obtained from a relaxed clock model.

### Bayesian phylogenetic analysis

SNPs, indels and mobile genetic elements (MGEs) from the index dog and staff members A, D and F were used to estimate the topologies of phylogenies of the strains found within these individuals. Separate analyses were performed for the two clades of MRSA found within staff member A. The data sets for each individual were divided into two partitions: (i) SNPs and (ii) indels and MGEs (Supplementary Data sets 2, 3 and 4). Evolutionary rates were estimated separately for these two partitions. A GTR model with a gamma distribution of rates over sites was used for the SNP partition. A two-state F81 model with no rate variation over sites was used for the indel/MGE partition. MrBayes was used to estimate the phylogenies^57^. No information about fixed sites was included in the models. Convergence and burnin were established through use of Tracer v1.6 (http://beast.bio.ed.ac.uk/Tracer). All MrBayes runs were well converged with estimated sample size (ESS) values >200. The topologies that resulted from the analysis of SNPs, indels and MGEs are described in Fig. 4. Trees represent consensus tree topology, with nodes present in <50% trees collapsed to polytomies, and rooting according to the Maximum likelihood (ML) topology.

### Calculation of diversity

The genetic diversity within populations of sequenced isolates was measured using standard population genetic statistics, namely, Watterson’s theta (a measure based on the number of segregating sites) and nucleotide diversity, pi, (the mean proportion of sites that differ between pairs of sequences^58^; Supplementary Fig. 2).

### Rarefaction and extrapolation

To estimate the number of additional variants that would be obtained from the further sequencing of isolates from a single host, a rarefaction and extrapolation analysis were performed using data from the nine swabs taken from the index dog^59^ (Supplementary Fig. 3). This analysis was performed using EstimateS 9.1.0 (EstimateS: Statistical estimation of species richness and shared species from samples. Version 9. http://purl.oclc.org/estimates)^60^.

### Copy number variation

To screen genomes for copy number variation, we used the cn.MOPS R package^61^ using the haplocn.mops algorithm adjusted for haploid genomes. All genomes were screened visually for regions with potentially changed copy number. Next, the mean read count per base was generated and an R script was then used to all regions to identify regions with an increase in copy number relative to the mean copy number of the entire genome. Regions identified to have an increased copy number were then manually inspected in the annotated genome sequence and by comparative genomics to identify the boundaries of mobile genetic elements. One region encoding a likely bacteriophage (φSa2_HO 5096 0412_, present at coordinates 1520142-1566315 in the reference genome HO 5096 0412 (ref. 13)) was identified to be variable in isolates in all three clades. To take into account the difference in coverage due to proximity to the origin of replication and terminus, the fold difference copy number of φSa2_HO 5096 0412_ sequences between isolates was calculated by dividing the mean read count of the entire region spanning φSa2_HO 5096 0412_ over the mean read count of 100 kb upstream and 100 kb downstream. Isolates with a φSa2_HO 5096 0412_ copy number ≥10% above or below the median (median copy numbers: 1.05, 1.36 and 1.19 for clade 1, 2 and 3, respectively) for the entire clade deemed to have a changed copy number (Supplementary Figs 4 and 5).

## Author contributions

G.K.P. contributed to the initiation and design of the study, the collection and processing of samples, the interpretation of data and wrote the manuscript. E.M.H. designed and conducted the bioinformatics analysis, analysed the data and wrote the manuscript. G.G.R.M. and J.J.W. carried out some phylogenetic analyses and contributed to the manuscript. J.H.W. collected clinical isolates and provided epidemiological data. M.T.G.H. conducted bioinformatic analysis and contributed to the manuscript. F.J.E.M. carried out microbiological isolations of the isolates. X.B. carried out experimental validation of mutations. G.K. contributed to the analysis and wrote coding scripts. S.R.H. wrote coding scripts, contributed to the analysis of the data and the manuscript. D.J.M. contributed to the study design and the manuscript. S.J.P. contributed to the interpretation of the data and the manuscript. M.E.H. contributed to the collection of isolates, provided epidemiological data and contributed to the manuscript. J.P. contributed to the initiation and design of the study, interpretation of the data and contributed to the manuscript. M.A.H. initiated and coordinated the study, contributed to the collection of isolates, analysed data and wrote the manuscript.

## Additional information

**How to cite this article:** Paterson, G. K. *et al*. Capturing the cloud of diversity reveals complexity and heterogeneity of MRSA carriage, infection and transmission. *Nat. Commun*. 6:6560 doi: 10.1038/ncomms7560 (2015).

## Supplementary Material

Supplementary Figures and Tables Supplementary Figures 1-5 and Supplementary Tables 1-2.

Supplementary Dataset 1 European short read archive accession numbers and assembly statistics for all isolates

Supplementary Dataset 2 Summary of clade 1 SNPS

Supplementary Dataset 3 Summary of clade 2 SNPS

Supplementary Dataset 4 Summary of clade 3 SNPS

Supplementary Dataset 5 Regions removed from the SNP alignment of clade 1 isolates

Supplementary Dataset 6 Regions removed from the SNP alignment of clade 2 isolates

Supplementary Dataset 7 Regions removed from the SNP alignment of clade 3 isolates
