# Supplementary material for: Capturing the cloud of diversity reveals complexity and heterogeneity of MRSA carriage, infection and transmission
Source: Nat Commun. 2015 Mar 27;6:6560. doi: 10.1038/ncomms7560 (PMC4389252; doi:10.1038/ncomms7560)
Supplement: Supplementary Figures and Tables — Supplementary Figures 1-5 and Supplementary Tables 1-2. [file ncomms7560-s1.pdf]

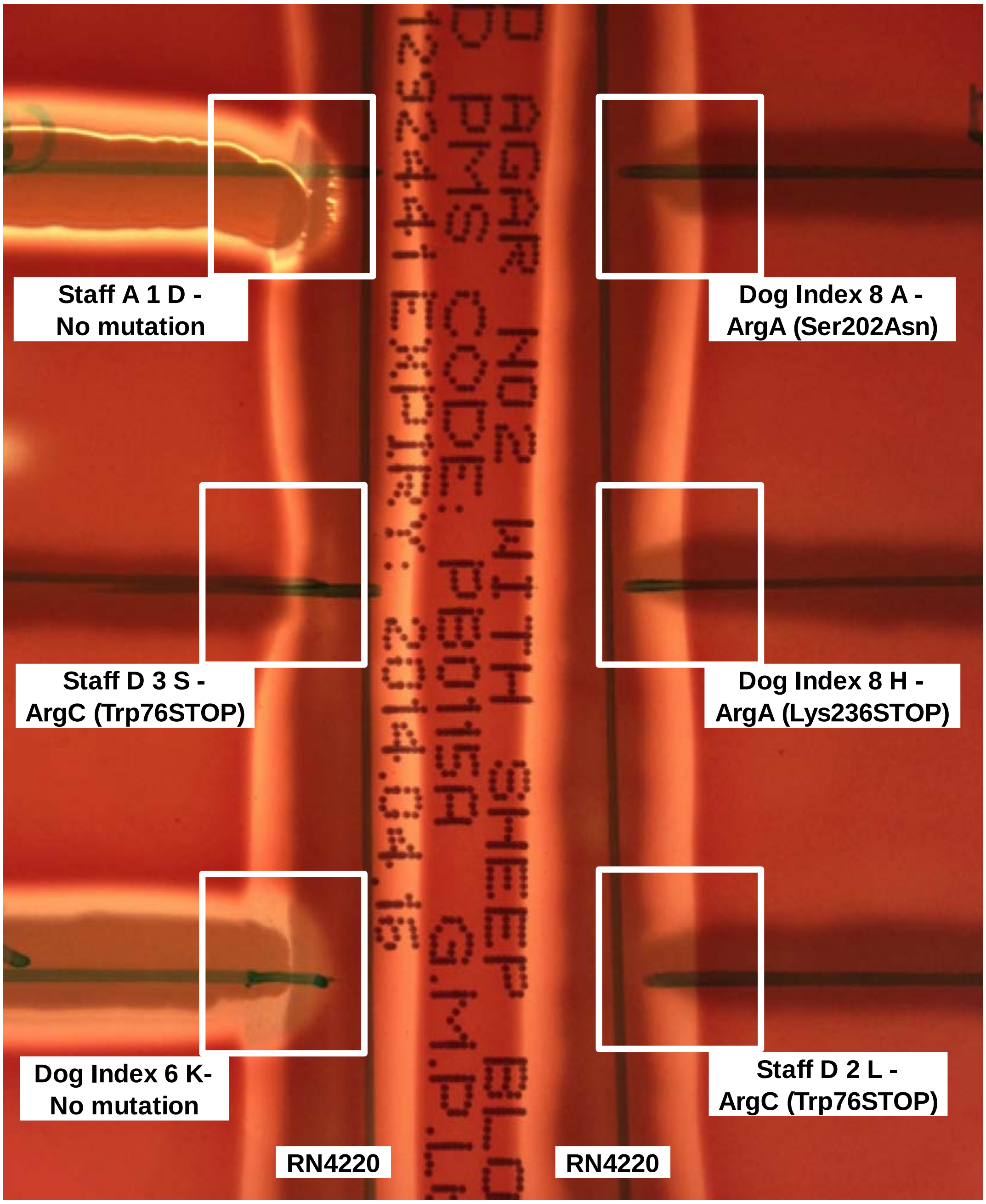

Supplementary Figure 1: Representative images of δ -haemolysis assay results. Image shows test strains streaked horizontally with streaks of RN4220 vertically. Boxed areas highlight area of synergistic haemolysis is present / absent between test isolates and RN4220.

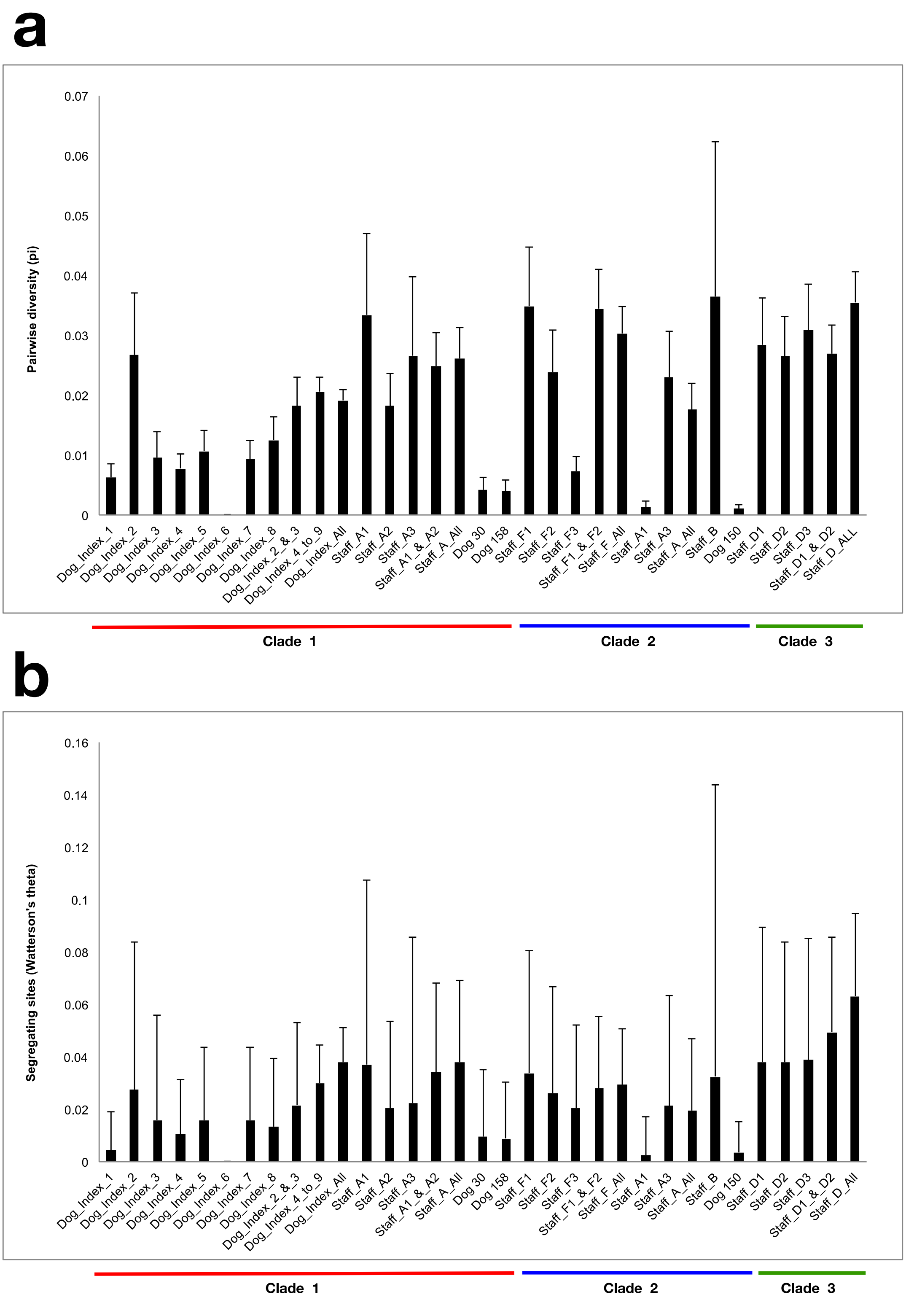

Supplementary Figure 2: Estimates of genetic diversity. (a) Pairwise diversity (pi). (b) Segregating sites (Watterson's theta). Error bars show standard error of the mean.

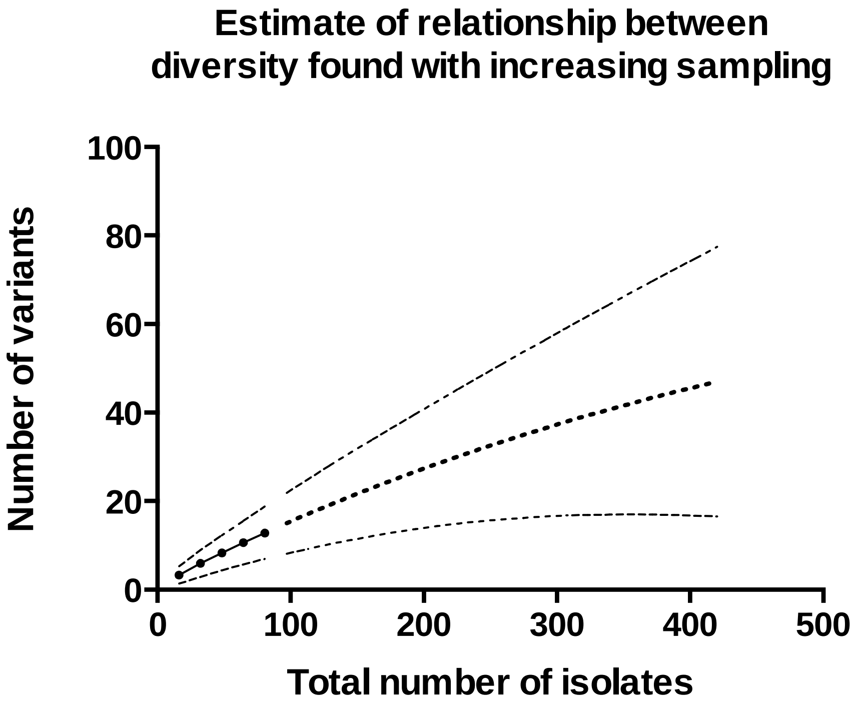

Supplementary Figure 3: Rarefaction analysis. The graph shows the relationship between the number of isolates obtained and the number of unique variants found within the sample. The solid black line with filled circles represents data obtained from the study. The dotted extrapolation of this line is the estimated increase in number of variants that would be obtained with an increased sample of isolates. The dashed lines represent the 95% confidence intervals.

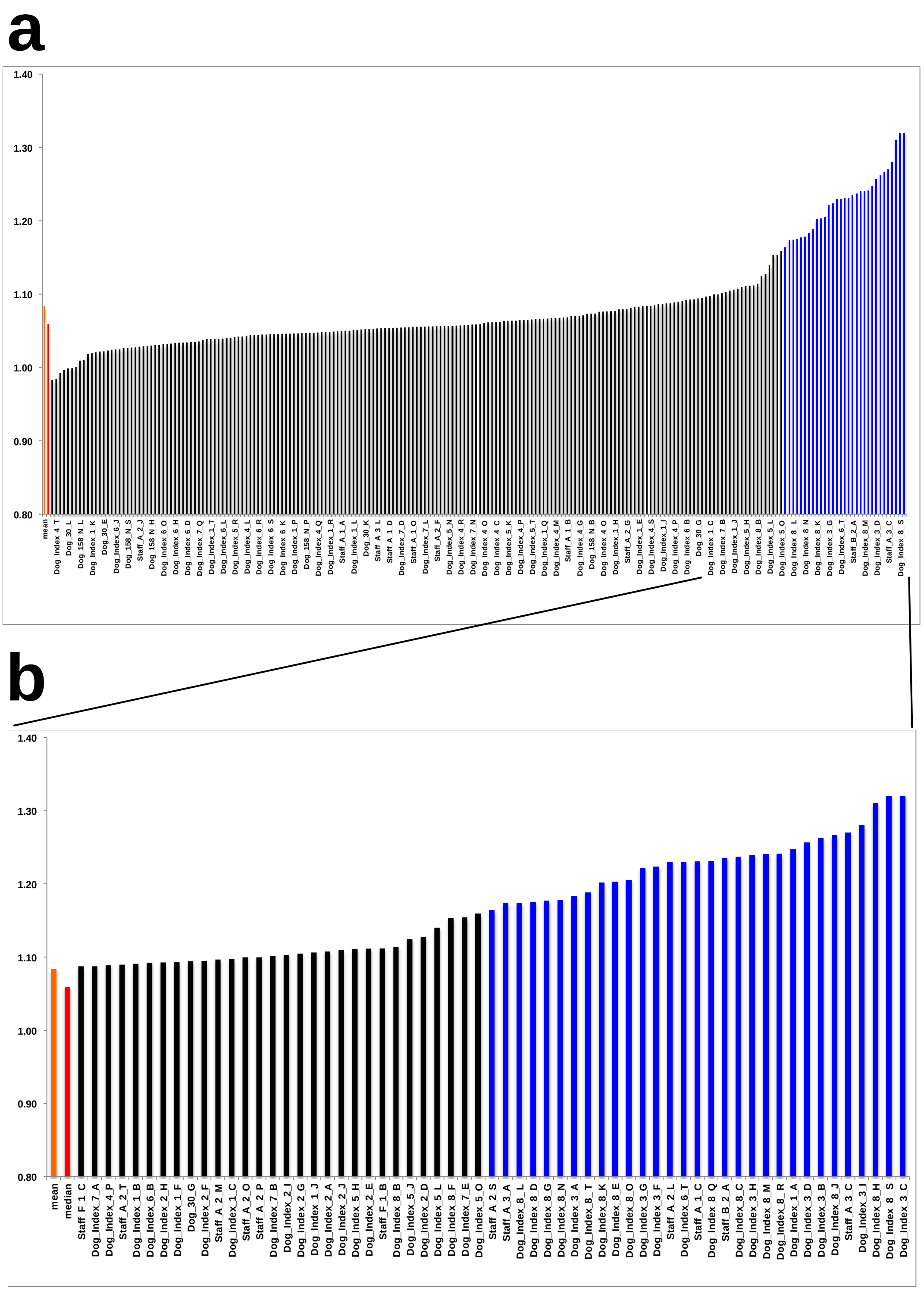

Supplementary Figure 4: Copy number variation of phage ϕSa2EMRSA15 in clade 1 isolates. (a) All isolates. (b) Zoomed in view of isolates with copy number increase. Values are the mean fold copy number normalised against 100kb up and downstream regions. The mean and median are highlighted in orange and red, respectively. Bars in blue represent isolates with a ≥10% increase in copy number realtive to the median copy number for all isolates in clade 1.

a

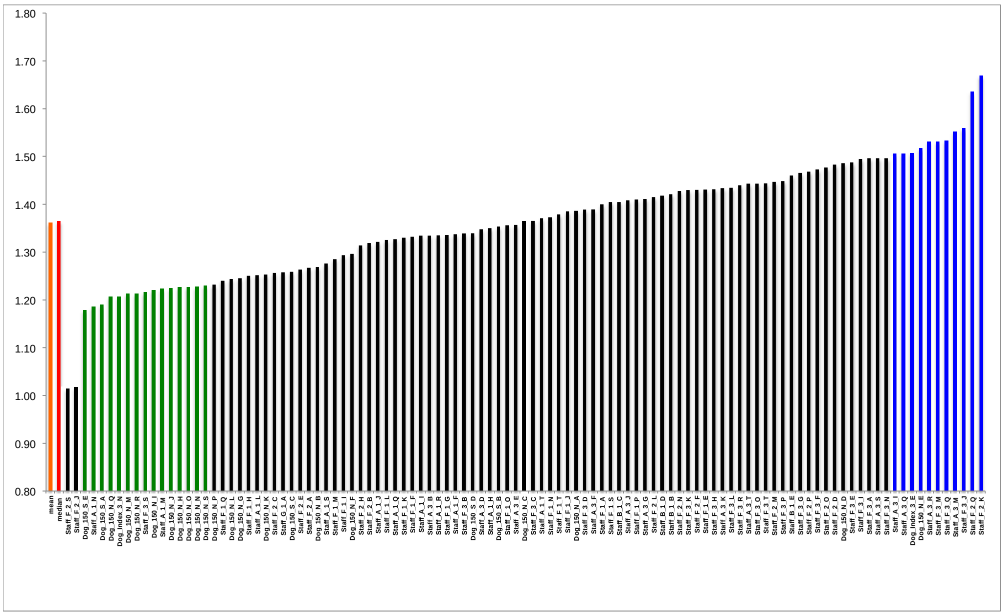

b

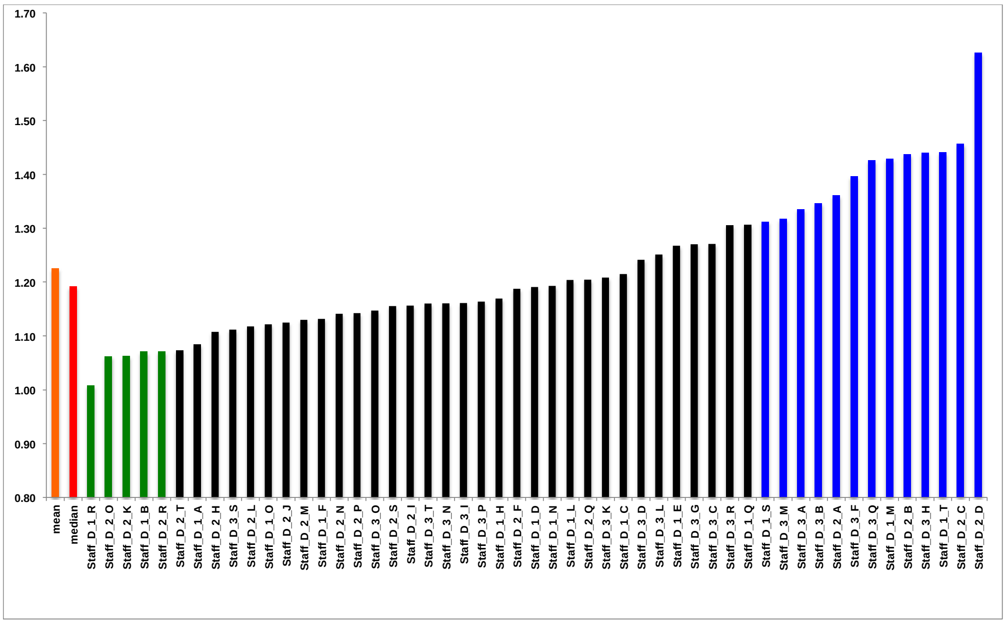

Supplementary Figure 5: Copy number variation of phage ϕSa2EMRSA15. (a) Clade 2 isolates. (b) Clade 3 isolates. Values are the mean fold copy of ϕSa2EMRSA15 normalised against 100kb up and downstream regions. The mean and median are highlighted in orange and red, respectively. Bars in blue and green represent isolates with a ≥10% increase or decrease in copy number realtive to the median copy number for all isolates in a clade, respectively.

Supplementary Table 1: Experimental measurement of the rate of spontaneous resistance to rifampicin

**Clade**	**Mutation rate**
**1**	$1.61(\pm0.46)\times10^{-7}$
**2**	$2.96(\pm0.30)\times10^{-7}$
**3**	$3.54(\pm0.38)\times10^{-7}$

Supplementary Table 2: Results of haemolysis assay

Isolate	Clade	δ -haemolysis	Mutation
Dog_Index_8_I	1	Negative	ArgA - Lys236STOP
Dog_Index_8_H	1	Negative	ArgA - Lys236STOP
Dog_Index_8_L	1	Negative	ArgA - Ser202Asn
Dog_Index_8_M	1	Negative	ArgA - Ser202Asn
Dog_Index_8_S	1	Negative	ArgA - Ser202Asn
Dog_Index_8_A	1	Negative	ArgA - Ser202Asn
Staff_A_1_D	1	Positive	none
Dog_Index_6_K	1	Positive	none
Dog_Index_7_O	1	Positive	none
Dog_Index_2_H	1	Positive	none
Dog_Index_7_S	1	Positive	none
Staff_D_2_L	3	Negative	ArgC - Trp76STOP
Staff_D_1_B	3	Negative	ArgC - Trp76STOP
Staff_D_3_S	3	Negative	ArgC - Trp76STOP
